# The Colour of the Hair

**Published:** 1912-01-20

**Authors:** 


### THE COLOUR OF THE HAIR.

There was a time in the old days of humoral pathology in which considerable importance was attached to colour of the hair or the shape of features as indications of deep-lying differences in physical qualities of the individual. Of recent years there has been a tendency to look upon outside appearances as more or less happy or unhappy accidents which may smooth or roughen the pathway of life according as the appearance is or is not pleasing, but as of no further consequence. Yet differences in colour must depend upon some chemical differences which can scarcely be only skin-deep. The fact at least appears to be generally accepted that in early periods of history there were considerable differences in physique and temperament between the dark-haired and fair-haired races. In most of the Western nations of to-day possibly the dark and the fair have become so mixed that we generally assume that if there were originally noteworthy differences between the dark and the fair, at the present time the intermingling of important characteristics has been so complete that colour may be ignored.

Complicated beings though we are, it is difficult to believe that the teachings of "Mendelism" may not in part be applicable to the human race, and that distinctive strains tend to persist. Dr. J. S. Macintosh has comparatively recently written a paper entitled in part "The Hygiene of the Blonde," in which he maintains that the susceptibility to various ailments differs in the dark and the fair. He also holds the opinion that the blonde is less able than the brunette to remain healthy and vigorous in large towns, and believes that the inability to adapt itself to conditions dependent upon modern industrialism is inherited from space-loving and sea-loving Saxon and Danish ancestors. Whatever truth there may be in the observation as to effect of town life upon blondes, the attitude adopted by our Saxon ancestors, as described by Tacitus, with regard to the surroundings of light and air, is interesting. There is undoubtedly remaining in a large proportion of the population an instinctive love of, it may almost be said a craving for, the comparatively free life of the open air. It is no doubt this instinctive yearning which explains the fascination which George Borrow's book, "Lavengro," may have for all sorts and conditions of men. Although Dr. Macintosh may be correct that in our mixed population this instinct is most evident in the fair, and that they suffer chiefly from "cramped surroundings," the most remarkable illustration of the power of human nature to withstand the influences and restraints of modern civilisation, curiously enough, occurs in a dark race—the gipsies. Borrow himself, we believe, was something of a mixture—possessed of abnormally light hair but dark eyes. The subject is an interesting one, and worthy of careful and extended observations; yet there may be some difficulty in determining in many instances what standard is to be adopted as to the demarcation between fairness and darkness of complexion. We all know the typical brunette or typical blonde, but a large percentage of the population has a non-descript-coloured hair, and it may be eyes a mixture of hazel and brown, or of brown and grey, which some might consider fair, but others dark. Apparently observations would require to be made under at least three headings.

Dr. Macintosh considers not only that the confined surroundings of the office or of the workshop tend most to a nervous breakdown in the fair, but he believes the fair also to be able less than the dark to withstand the deleterious effects of low-lying and damp districts. He states that "both in this country and in Holland the districts with a marshy or cold clay wet soil are inhabited by brunettes and avoided by blondes." On more than one ground it appears therefore that Dr. Macintosh believes—at least so far as modern conditions are concerned—in the physical superiority of the brunette, and thus agrees with the foreign authority, described in a daily paper as "French savant." The view is not altogether new at least from one standpoint. Several observers have expressed the opinion for some years that the dark are increasing in numbers at the expense of the fair, which, if true, may be taken as an evidence of superiority in some form of vitality, either that of fertility or of power of resistence against disease.

